# C/EBP Homologous Protein (CHOP) Deficiency Aggravates Hippocampal Cell Apoptosis and Impairs Memory Performance

**DOI:** 10.1371/journal.pone.0040801

**Published:** 2012-07-16

**Authors:** Chang-Mu Chen, Cheng-Tien Wu, Chih-Kang Chiang, Bor-Wu Liao, Shing-Hwa Liu

**Affiliations:** 1 Division of Neurosurgery, Department of Surgery, National Taiwan University Hospital and National Taiwan University, College of Medicine, Taipei, Taiwan; 2 Institute of Toxicology, College of Medicine, National Taiwan University, Taipei, Taiwan; 3 Department of Integrated Diagnostics & Therapeutics, National Taiwan University Hospital and National Taiwan University, College of Medicine, Taipei, Taiwan; 4 Department of Otolaryngology, Cardinal Tien Hospital, Yung Ho Branch, New Taipei City, Taiwan; Duke University Medical Center, United States of America

## Abstract

Neurodegenerative disorders are growing burdens in modern societies because of increased life expectancy. Most neurodegenerative disorders commonly possess a similar neuropathological feature - the accumulation of abnormal protein aggregates or inclusions (misfolded proteins) in the brain. One of the main functions of endoplasmic reticulum (ER) is to initiate proper protein folding to facilitate protein secretion through the induction of unfolded protein response (UPR). C/EBP homologous protein (CHOP) induction has been demonstrated to be a signaling event underlying ER stress-induced cell apoptosis. In this study, we explored the role of CHOP in the hippocampal cell apoptosis and memory performance injury under an induced ER stress condition. Adult male wild type (C57BL/6J) and CHOP knockout (CHOP−/−) mice were intracerebroventricularly injected with tunicamycin. Tunicamycin can induce ER stress and cell apoptosis in mouse hippocampus. Compared with wild type mice, CHOP−/− mice showed an enhanced hippocampal cell apoptosis, worse performance in memory-related behavioral tests, and attenuated IRE-1 expression under tunicamycin treatment. The aggravated cell apoptosis and worse memory performance in CHOP−/− mice might be due to the deficiency of CHOP protein resulted in the impaired adaptive/pathological transcriptional response, the decreased IRE-1 and XBP-1 expressions, and the increased JNK phosphorylation to cope with ER stress. Taken together, these results suggest that CHOP may play a protective role in the hippocampal cell apoptosis and impairment of memory performance.

## Introduction

Neurodegenerative disorders are growing burdens in modern societies because of increased life expectancy. Most neurodegenerative disorders commonly possess a similar neuropathological feature - the accumulation of abnormal protein aggregates or inclusions (misfolded proteins) in the brain. Abnormal protein aggregation impede many essential cellular functions, and thus lead to neuronal loss and caused various neurological impairments in these diseases [1]. The common neurodegenerative diseases include Parkinson’s disease (PD), amyotrophic lateral sclerosis (ALS), Alzheimer’s disease (AD), Huntington’s disease (HD), and many others. Endoplasmic reticulum (ER) is an intracellular organelle, and one of its main functions is to initiate proper protein folding to facilitate protein secretion. To achieve this goal, a complex network of protein chaperones, foldases, and co-factors are present at the ER lumen to catalyze the folding and maturation of proteins, and to prevent their abnormal aggregation or misfolding. If there are disturbances occurred in ER homeostasis, the accumulation of abnormally folded proteins will appear in the ER lumen, and lead to a condition known as ER stress. In ER stress, the unfolded protein response (UPR) will be triggered. UPR is an adaptive reaction that increases the cell’s capacity to produce properly folded proteins and decreases the unfolded protein load [2]. Once UPR is activated, the expression of different proteins with functions in almost every aspect of the secretory pathway will be affected. These functions include folding, quality control, protein entry into the ER, ER-associated degradation, autophagy-mediated degradation, and many others [3].

There are three main types of ER stress sensors that can activate UPR signaling responses. These sensors are ER resident transmembranous signaling proteins, which include double-stranded RNA-activated protein kinase-like ER kinase (PERK), activating transcription factor 6 (ATF6), and inositol requiring kinase 1 (IRE1). The function of these sensor proteins is to transduce the information about the protein folding status at the ER lumen to the nucleus and cytosol through controlling the expressions of specific transcription factors and other rapid effects on protein synthesis [1]. Prolonged ER stress will ultimately lead to cell apoptosis. Several regulators have been identified to mediate cell apoptosis, including the BCL-2 family of proteins [2], [4] and activation of ASK1 and JNK [5], [6]. In addition, sustained PERK signaling is proposed as a pro-apoptotic effector, and such effect is possibly through the induction of C/EBP homologous protein (CHOP)/GADD153 and the BCL-2 family member BIM and PUMA [7]–[10]. CHOP is a 29 kDa protein with 169 (human) or 168 (rodents) amino-acid residues. CHOP is also known as growth arrest and DNA damage inducible gene 153 (GADD153), DNA-damage-inducible transcript 3 (DDIT3) and C/EBPζ [11]. Induction of CHOP may trigger ER stress-induced apoptosis, and the involvement of CHOP-mediated apoptosis has been demonstrated in various diseases, including diabetes, neurodegenerative diseases, brain ischemia, and some cardiovascular diseases [12]. However, the role of CHOP in neurological disorders has not been thoroughly investigated. In this study, we try to investigate the role of CHOP in the hippocampal cell apoptosis and memory performance impairment in a mouse model of CHOP knockout with ER stress induction.

## Materials and Methods

### Animals

*Chop* deficiency mice (C57BL/6 background) were purchased from Jackson Laboratories (Bar Harbor, ME). Adult male mice (wild type (C57BL/6) and CHOP knockout (Chop−/−), about 18–25 g, were used in this study. The Animal Research Committee of College of Medicine, National Taiwan University, approved and conducted the study in accordance with the guidelines for the care and use of laboratory animals. The animals were take care with humane and regard for alleviation of suffering. Mice were housed in a room at a constant temperature of 22±2°C with 12 h light-dark cycles.

**Figure 1:**
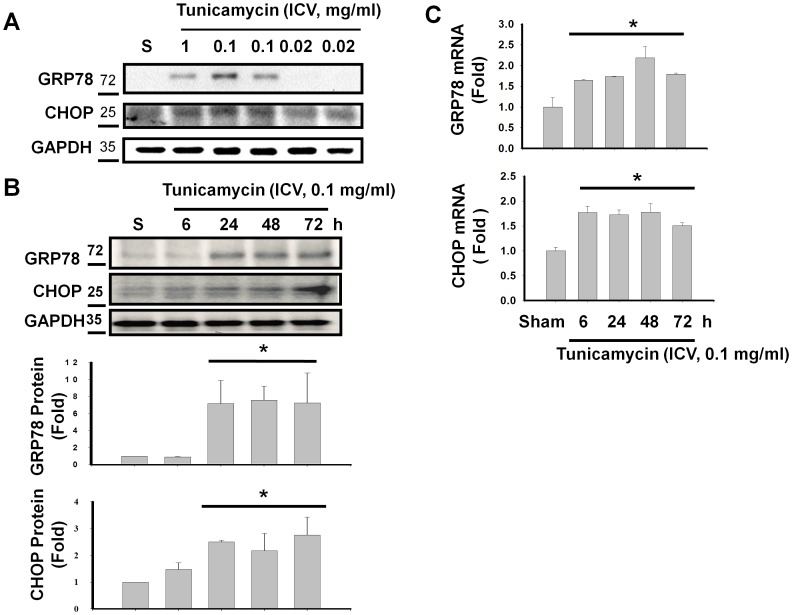
Expressions of ER stress-related molecules GRP78 and CHOP in the mouse hippocampus with or without tunicamycin treatment. C57BL/6J mice were intracerebroventricular injected with tunicamycin (0.02–1 mg/ml) at various time course (6–72 h). In A, the levels of GRP78 and CHOP proteins in hippocampus were determined by Western blotting 24 h after tunicamycin injection in a dose-dependent manner. Representative images of three independent experiments are shown. In B, the levels of GRP78 and CHOP proteins from hippocampus were determined by Western blotting after tunicamycin (0.1 mg/ml) injection in a dose-dependent manner. In C, the levels of GRP78 and CHOP mRNAs from hippocampus were determined by real-time PCR after tunicamycin (0.1 mg/ml) injection in a time-dependent manner. In B and C, quantification of proteins and mRNA levels were shown. Data are presented as mean ± S.E.M. (n = 3). *P<0.05 as compared with sham-control.

### Genotyping of CHOP Knockout Mice

The genotype for *Chop* was determined by PCR. The following primers were used: the sense primer was 5′-ATGCCCTTACCTATCGTG-3′, and the antisense primers were 5′-AACGCCAGGGTTTTCCCAGTCA-3′ (derived from the neomycin resistance gene), and 5′-GCAGGGTCAAGAGTAGTG-3′ (derived from the *Chop* gene). The thermal cycle reaction was performed as the Jackson Lab announces process: 94°C for 3 min, followed by 35 cycles at 94**°**C for 30 s, 61**°**C for 1 min, 72°C for 1 min, and 72°C for 2 min. The wild-type and recombinant alleles each yielded the transcripts of 545 and 320 bps, respectively. The CHOP protein was determined by the Western blotting.

### Intracerebroventricular Injection of Tunicamycin

Weight and rectal temperature of each mouse were recorded before the surgical procedure. Anesthesia was induced with 5% chloral hydrate (400 mg/kg). Each mouse was mounted on a stereotaxic frame. Tunicamycin prepared in phosphate-buffered saline was injected into the cerebral ventricle (stereotaxic coordinates PA-1.0 mm, lateral-1.5 mm from bregma, and ventral-2.0 mm relative to dura) of mouse as previously described [13]. All mice received the same procedure except the same amount of normal saline was injected in the sham operation group. After injections, mice were placed in a humidified, thermo-regulated chamber maintained at 31°C and were returned to their cages after full recovery from anesthesia. Throughout the experimental procedure, mouse rectal temperature was monitored and maintained at 37.0±0.5°C.

### Real Time-PCR

Real-time PCR was used the reverse transcriptase reagent mix (Promega, San Luis, CA, USA). Briefly, total RNA (10 µg) was reverse transcribed in the total volume of 40 µl reagent mix. Following to incubate the mixture on 37°C for 90 min and denature on 95°C for 15 min, the real-time PCR was performed in the Bio-Rad iQ5 system. The RT reaction products were diluted to 5 fold of GAPDH, and 10 fold of 18 S rRNA, respectively. 1 µl aliquots were used as the template for RT-PCR detection with SYBR Green system (Promega, San Luis, CA, USA). The primer sets for mouse GRP78 (forward, GAAAGGATGGTTAATGATGCTGAG; reverse, GTCTTCAATGTCCGCATCCTG), CHOP (forward, ATGCCCATCTTCTGCTTGTCA; reverse, CCTTGTAGTTGTGGGTCTTGT), GAPDH (forward, TGGCACAGTCAAGGCTGAGA; reverse, CTTCTGAGTGGCAGTGATGG) and 18 S rRNA (forward, AGTCCCTGCCCTTTGTACACA; reverse, CGATCCGAGGGCCTCACTA). Total DNA was normalization by the 18 S rRNA and GAPDH signals amplification in each separate experiment.

### Western Blotting

Hippocampal tissues were lysed by the ice-cold RIPA buffer and supplemented with protease inhibitor mixture (Santa Cruz Biotechnology, Santa Cruz, CA, USA). The equal quantification protein was subjected loading to the SDS-PAGE. The transfer membrane was first incubated for 1 h in 50 mM Tris-HCl, pH 7.5, 150 mM NaCl, 0.1% Tween 20 (TBST buffer) and 5% skimmed milk and then overnight at 4°C with primary antibodies: anti-CHOP, anti-GRP78, anti-XBP-1, anti-phospho-JNK, anti-JNK (1∶1000; Santa Cruz Biotechnology, Santa Cruz, CA, USA), anti-phospho-IRE1, anti-IRE1, anti-procaspase-9 (1∶2000; Cell Signaling Technology, Danvers, MA, USA), anti-procaspase 12 (1∶1000; Abcam, Cambridge, MA, USA), and anti-GAPDH (1∶5000; Sigma-Aldrich Corp, St. Louis, MO, USA). After washing, the filter was incubated with horseradish peroxidase-conjugated secondary antibodies (1∶5000; Jackson ImmunoResearch, Espoo, Finland), followed by detection using enhanced chemiluminescence (Pierce, Helsinki, Finland). Quantification was performed by GelDoc (Bio-Rad, Espoo, Finland).

**Figure 2:**
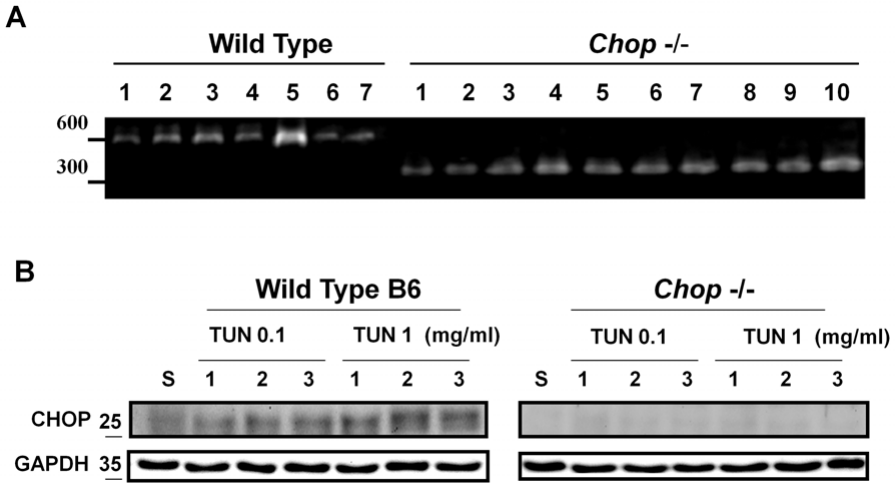
*Chop* gene and protein expressions in the hippocampus isolated from wild type and *chop* knockout mice. *Chop* genome typing and protein expression in the hippocampus isolated from C57BL/6J mice and *chop*−/− mice 1 day after intracerebroventricular injection of tunicamycin (0.1 and 1 mg/ml) were detected by the PCR (A) and Western blotting (B), respectively. Representative images of three independent experiments are shown.

**Figure 3:**
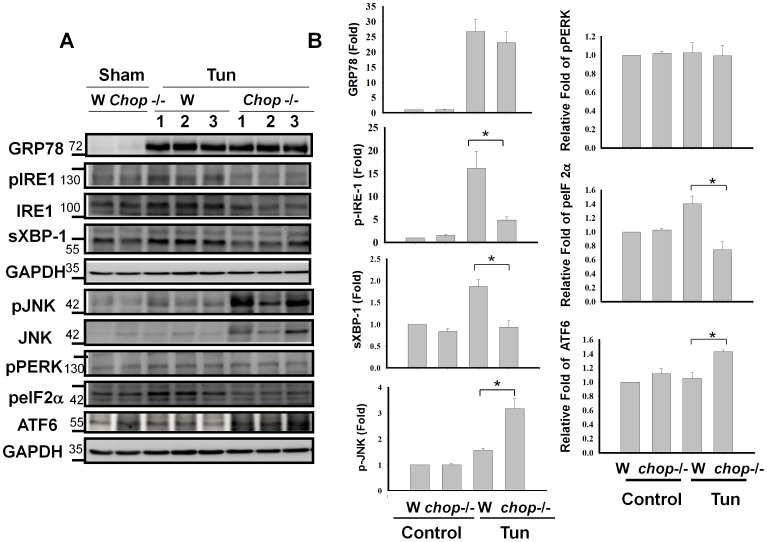
Expressions of ER stress-related molecules GRP78, IRE-1, phospho-IRE-1, XBP-1, JNK, phospho-JNK, phospho-PERK, phospho-eIF2α, and ATF6 in the hippocampus isolated from wild type and *chop* knockout mice. The expressions of GRP78, IRE-1, phospho-IRE-1, XBP-1, JNK, phospho-JNK, phospho-PERK, phospho-eIF2α, and ATF6 in the hippocampus isolated from C57BL/6J and *chop*−/− mice 5 days after intracerebroventricular injection of tunicamycin were performed by the Western blotting (A). Quantification of protein expressions were shown in B. Data are presented as means ± S.E.M. for three independent experiments. Each data was performed in triplicate. *P<0.05 as Tun+Wild Type versus Tun+*chop−/−* mice.

### Terminal Deoxynucleotidyl Transferase-mediated Biotinylated UTP Nick end Labeling (TUNEL) Staining

Frozen 4 µm were air dried for 30 min at 60°C, cleared in xylene, and mounted in dibutyl phthalate xylene. Subsequently, sections were immersed in 1% sodium hydroxide and 80% ethanol for 5 min, following rinse with 70% ethanol for 5 min, and then immersed in distilled water. After washing for 3 times, the sections were stain with Terminal deoxynucleotidyl transferase-mediated biotinylated UTP nick end labeling (TUNEL) staining (Promega Corporation, Madison, WI, USA) and the procedure was done as the manufacturer procedure. Following the TUNEL stain, the sections were rinsed in phosphate-buffer saline (PBS) and incubated with ammonium chloride for 20 min. Finally, Hoechst 33258 (1 µg/ml, Sigma-Aldrich Corp, St. Louis, MO, USA) counter stain was performed. The slides were washed and mounted with Mounting medium (Dako Inc, Carpinteria, CA, USA). The TUNEL-positive cells were counted in 20 randomly selected visual fields at the same levels in control and treated mice under 200× magnification.

### Immunofluorescence Staining

Neuronal tissue were isolated and fixed with 10% buffered formaldehyde. Subsequently, 5-µm-thick paraffin sections were prepared for immunofluorescence staining as below. Briefly, sections were deparaffinized with xylene and rehydration with 90%, 85%, 70% and 50% alcohole for 5 min for each step. After boiling the tissue sections in citrated buffer (PH 6.0) for 30 min, the sections were blocking with 5% goat serum for 1 hour and following incubation the neuronal marker Neu-N (1∶20, Millipore Corp., Billerica, USA) antibody and anti-mouse FITC secondary antibody (1∶200, Sigma-Aldrich Corp., St, Louis, USA). Finally, sections were co-labeled with 4′,6-diamidino-2-phenylindole (DAPI) and mounted with Vectashield (VectorLab, Buringame, CA, USA). The fields were focus on the Hippocampus region and used with 40× magnification.

### Water Maze

A circular pool (diameter 1.7 m, rim 50 cm high) for water maze was used. The pool was painted black and filled with water to a depth of 30 cm. In one of its quadrants, a hidden black escape platform (12×12 cm) was placed, and its top lay 2 cm beneath the water surface. The experiment was initiated by placing the mice in one of the three other quadrants near the wall of the pool. In each test, mice were allowed to search for the escape platform. Time to target is the time spent in finding the hidden platform. Time in target quadrant is the time stayed in the quadrant of the hidden platform.

**Figure 4:**
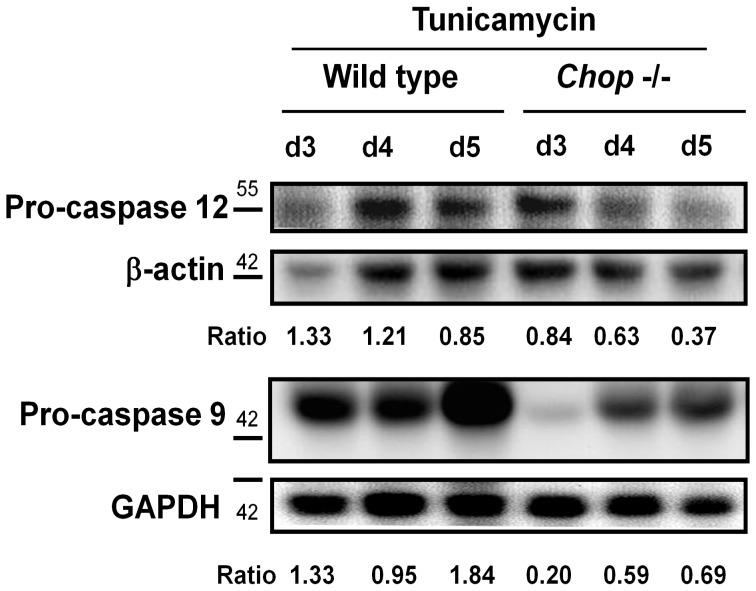
Expressions of pro-caspases 9 and 12 in the hippocampus isolated from wild type and *chop*−/− mice. Tunicamycin (0.1 mg/ml) were intracerebroventricular injected in the hippocampus of C57BL/6J and CHOP−/− mouse for 3–5 days. Pro-caspase 12 and pro-caspase 9 expressions were presented by the Western blotting from day 3 to day 5 after tunicamycin injection. Representative images of three independent experiments are shown.

**Figure 5:**
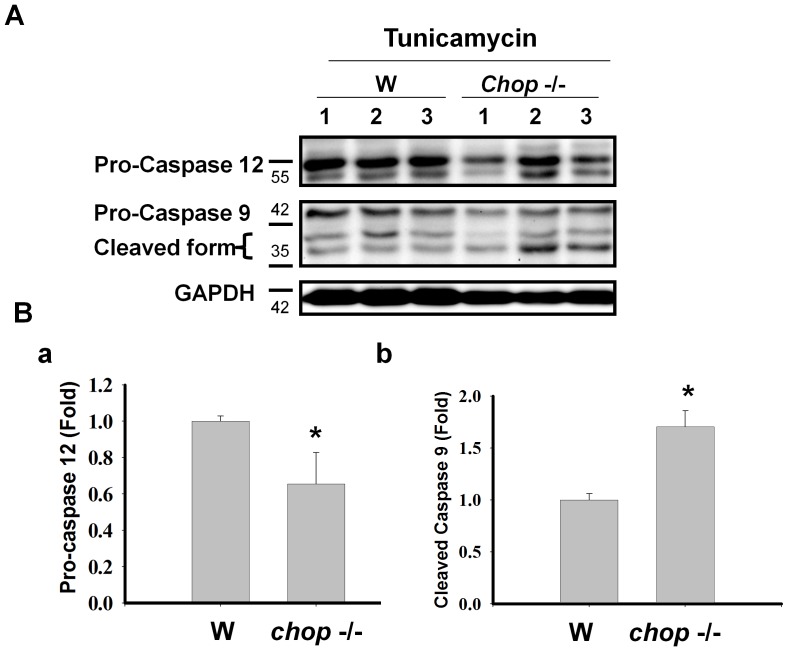
Expressions of pro-caspases/caspases 9 and 12 in the hippocampus isolated from wild type and *chop*−/− mice. (A) Tunicamycin (0.1 mg/ml) was intracerebroventricular injected in the hippocampus of C57BL/6J and CHOP−/− mouse for 3–5 days. Pro-caspase 12 and pro-caspase 9 expressions were presented by the Western blotting from day 3 to day 5 after tunicamycin injection. Representative images of three independent experiments are shown. (B) The expressions of pro-caspase 12, pro-caspase 9, and cleaved caspase 9 in the hippocampus of 57BL/6J and *chop*−/− mice at day 5 after treatment with tunicamycin (0.1 mg/ml) were detected by the Western blotting. Quantification of pro-caspase 12 (B-a) and cleaved caspase 9 (B-b) were shown. Data are presented as mean ± S.E.M. for each group (n = 3). *P<0.05 as Tun+Wild Type versus Tun+*chop−/−* mice.

**Figure 6:**
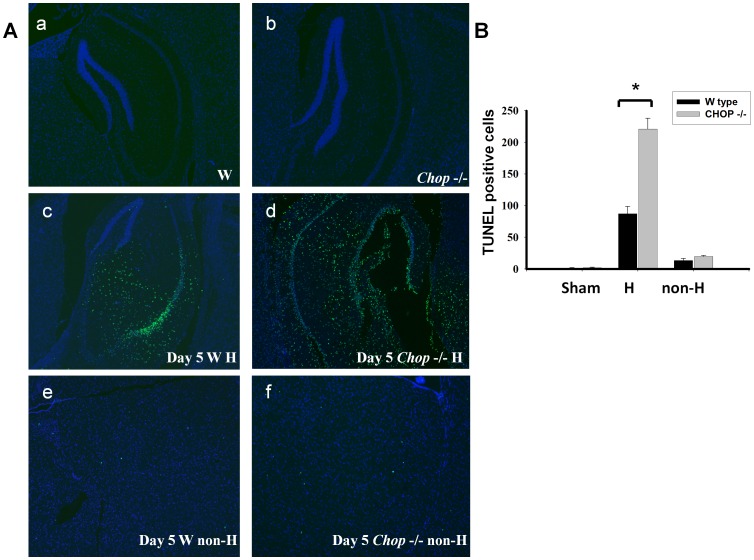
Cell apoptosis in the hippocampus or cortex regions were isolated from wild type and *chop*−/− mice. Tunicamycin (0.1 mg/ml) were injected in the hippocampus of wild type C57BL/6J and CHOP−/− mouse. Apoptotic cells were performed by the TUNEL staining 5 days after tunicamycin injection. The staining of neuron nuclei in normal control hippocampus was shown on top (A), and the TUNEL positive cells from hippocampus or cortex (B) of wild type C57BL/6J and *chop*−/− mouse were quantified (C). Data are presented as mean ± S.E.M. for each group (n = 4). **P<0.01 as Tun+wild type versus Tun+*chop−/−* mice. H: hippocampus; C: cortex.

**Figure 7:**
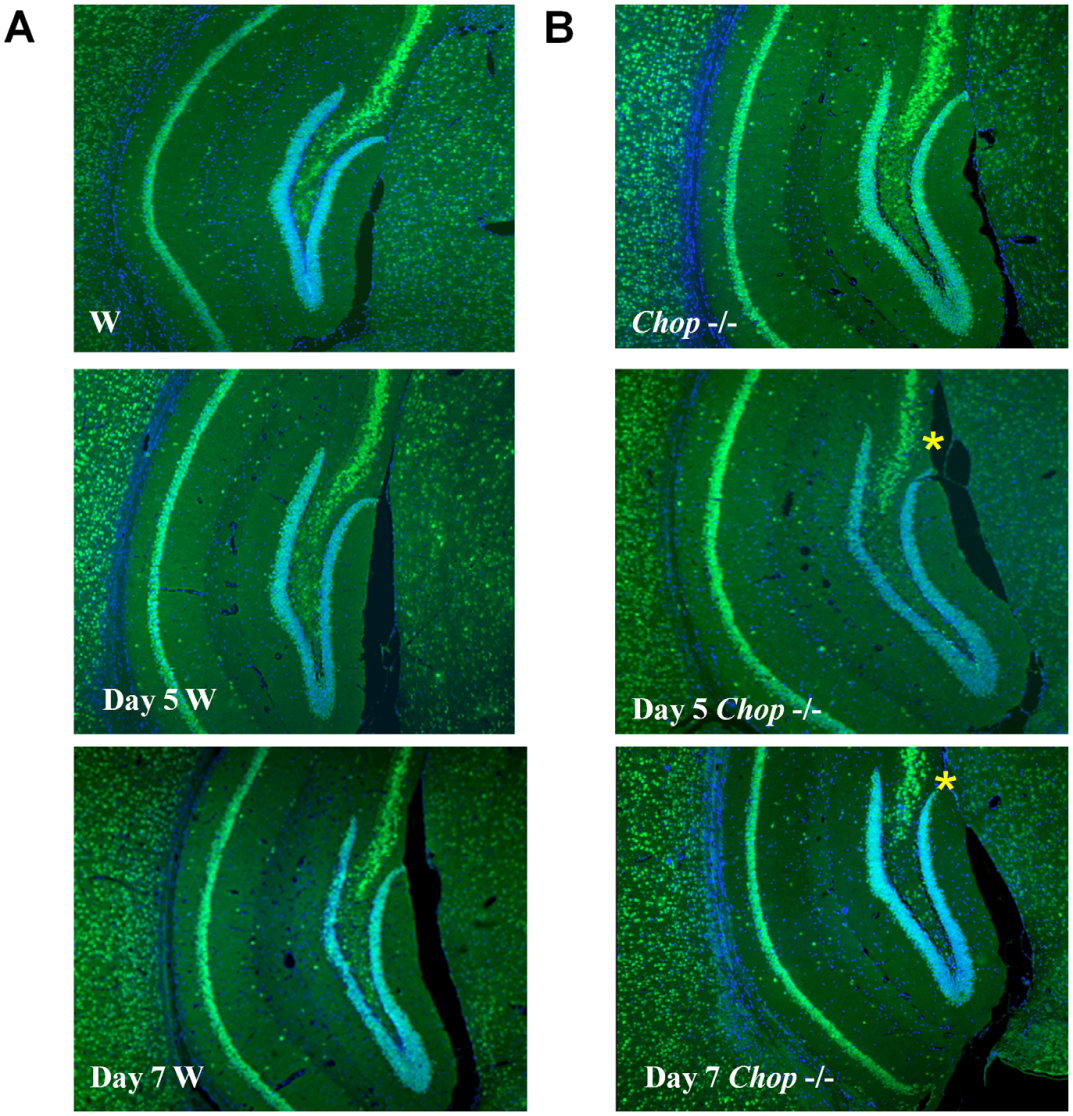
Neuronal living cells in hippocampus of wild type and CHOP−/− mice. Tunicamycin (0.1 mg/ml) were intracerebroventricular injected in the hippocampus of wild type C57BL/6J (W; A) and CHOP−/− (B) mouse for 5–7 days. Neuronal cells were identified by immunofluorescence staining with Neu-N (green fluorescence). Representative images of three independent experiments are shown. The star symbol indicates that the neuronal living cells are markedly decreased in hippocampus of CHOP−/− mice.

### Passive Avoidance Test

Learning and memory in the wild type and *chop* deficiency mice were examined by using the step-down type of passive avoidance task. The test apparatus (Huaibei Zhenghua Biologic Apparatus Facilities Ltd.Co., Suixi, China) consisted of a chamber (12×12 18 cm) having a grid floor with a wooden platform (5×5×4.5 cm) at the right lower corner of the grid floor. During the experimental period, the chamber was illuminated with a 15 W lamp. Mice were trained to learn avoiding electric stimulus (0.25 mA, 100 V). Each mouse was placed on the grid floor with back against the platform, and an electric shock was delivered to the grid floor. The time taken by the mouse to first jump on the platform was recorded as learning latency. A retention test was performed after 24 h. Each mouse was against placed on the platform. The duration of time before it stepped down to the grid floor was measured as the avoidance latency.

### Statistical Analysis

Statistical comparisons between study groups were performed using one-way ANOVA test followed by post hoc multiple comparison with Dunnett’s test. *P* values of less than 0.05 were considered to be statistically significant.

**Figure 8:**
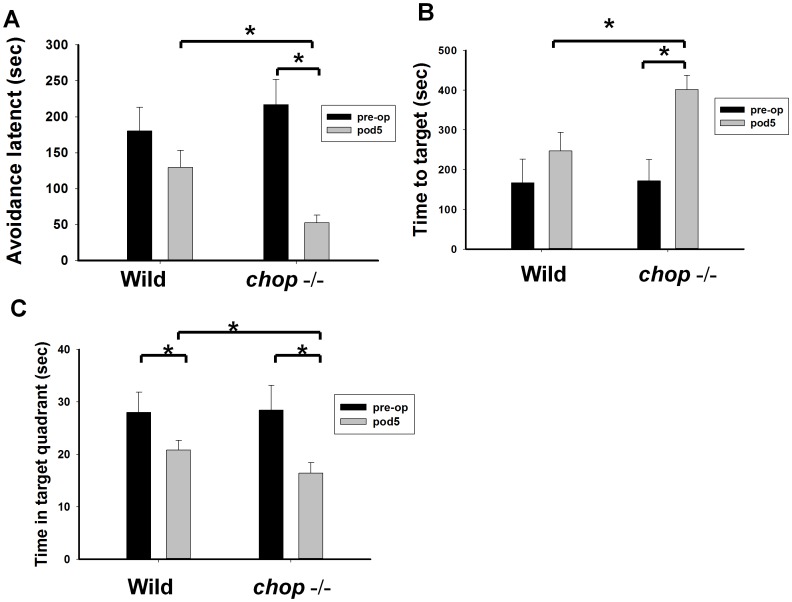
Behavior analysis in wild type and CHOP−/− mice with or without tunicamycin treatment. (A). Passive avoidance was performed 1 day before and 5 days after administration of tunicamycin (0.1 mg/ml) in wild type and CHOP−/− mice. The avoidance latency is significantly decreased in *chop*−/− mice than in wild type mice 5 days after tunicamycin administration. (B). Water maze was performed 1 day before and 5 days after administration of tunicamycin (0.1 mg/ml) in wild type and *chop*−/− mice. The time spent in finding the target platform is significantly increased in *chop* −/− mice than in wild type mice 5 days after tunicamycin administration. (C). In water maze, the time spent in the target quadrant is significantly decreased in *chop*−/− mice than in wild type mice 5 days after tunicamycin (0.1 mg/ml) administration. Data are presented as mean ± S.E.M. for each group (n = 5). *P<0.05 as compared with wild type mice or *chop−/−* mice without tunicamycin.

## Results

### Expressions of ER Stress-related Molecules in the Hippocampus of Wild Type and CHOP Knockout Mice

Firstly, tunicamycin was intracerebroventricularly injected into lateral ventricle of normal C57BL/6J mice at various concentrations (0.02, 0.1, and 1 mg/ml), and mouse hippocampal tissues were harvested 24 h after tunicamycin injection. The expressions of ER stress-related molecules GRP78 and CHOP were markedly induced by tunicamycin at the concentrations of 0.1 and 1 mg/ml (Fig. 1A). It appeared that ER stress can be induced when the injected concentration of tunicamycin is above 0.1 mg/ml, and the dosage of 0.1 mg/ml is used in later experiments. Moreover, the time courses of induction in ER stress-related molecules GRP78 and CHOP were examined by Western blotting and real time-PCR. As shown in Figure 1, the induction of GRP78 and CHOP mRNA expressions started at 6 h and maintained to 72 h after tunicamycin injection, and the induction of GRP78 and CHOP protein expressions started at 24 h and maintained to 72 h after tunicamycin injection.

We next performed genotyping and Western blotting for CHOP in the hippocampus of wild type C57BL/6J and CHOP−/− mice 24 h after tunicamycin injection. As shown in figure 2, the wild type and recombinant alleles each yielded the transcripts of 545 and 320 bps, respectively, and the protein expressions were not detected in CHOP−/− mice (Fig. 2). We further performed Western blotting for GRP78, IRE-1, XBP-1, JNK, PERK, eIF2α, and ATF6 expressions in the hippocampus of wild type C57BL/6J and CHOP−/− mice 5 days after tunicamycin injection. No significant difference was noted in the expression of GRP78 between wild type and CHOP−/− mice (Fig. 3). However, the expressions of IRE-1, phospho-IRE-1, and XBP-1 were significantly decreased, and the expression of phospho-JNK was significantly increased in CHOP−/− mice (Fig. 3A and 3B–a). There was no significant difference in PERK activation between wild type and CHOP−/− mice under tunicamycin treatment, but the phospho-eIF2α expression was decreased in CHOP−/− mice. Moreover, the ATF6 expression was increased in hippocampus of tunicamycin-treated CHOP−/− mice. (Fig. 3A and 3B–b).

### Enhancement of Hippocampal Cell Apoptosis in CHOP−/− Mice

We further investigated the activation of caspases 9 and 12 in the hippocampus of wild type and CHOP−/− mice 3 to 5 days after tunicamycin injection. As shown in Figure 4, the activations of caspases 9 and 12 (the decrease in pro-caspases) in the hippocampus were more pronounced in CHOP−/− mice than in wild type mice after tunicamycin administration. After quantification, the activation of caspase 12 and 9 was significantly increased in CHOP−/− mice on day 5 after injection of tunicamycin (Fig. 5). These results showed that CHOP−/− mice had a more pronounced activation of apoptotic signaling than wild type after induction of ER stress (tunicamycin injection). Furthermore, 5 days after injection of tunicamycin, the hippocampal tissues from wild type and CHOP−/− mice were prepared for TUNEL staining. As shown in Figure 6, the number of TUNEL-positive cells was significantly increased in the hippocampus of CHOP−/− mice as compared with wild type mice. However, a small number of TUNEL-positive cells were found in brain outside of hippocampus of tunicamycin-treated wild type and CHOP−/− mice; there was no statistically significant difference between wild type group and CHOP−/− group (Figs. 6A–e,f and 6B). The apoptotic cells seem to include both neuronal and glial cells. In addition, for identification of neuronal living cells, we performed immunofluorescence staining with neuronal marker Neu-N in hippocampus 5 and 7 days after tunicamycin injection. The surviving neuronal cells were more obvious in wild type than in CHOP−/− mice (Fig. 7).

### Behavior Analysis in Wild Type and CHOP−/− Mice with or Without Tunicamycin Treatment

Next, we performed the behavior analysis including passive avoidance test and water maze test in wild type and CHOP−/− mice 5 days after tunicamycin injection. In passive avoidance test, the avoidance latency was significantly shorter in CHOP−/− mice than in wild type mice (Fig. 8A). In water maze test, the average time spent to find the target platform was significantly increased (Fig. 8B), and the time spent in the target quadrant was significantly decreased in CHOP−/− mice (Fig. 8C). These findings indicated that CHOP−/− mice suffered from more severe damages in memory-related performance tests after injection of tunicamycin.

## Discussion

In this study, we demonstrated for the first time the role of CHOP in the hippocampal cell apoptosis and memory performance impairment in a mouse model. CHOP knockout mice were utilized to elucidate the role of CHOP in the hippocampus. Intracerebroventricular injection of tunicamycin induced ER stress and both neurons and glial cell apoptosis in hippocampus. Compared with wild type mice, CHOP−/− mice showed the increased hippocampal cell apoptosis, the worse performance in memory-related behavioral tests, and the attenuated phosphorylated IRE-1 and eIF2α expressions and the enhanced ATF6 and JNK expressions in hippocampus. These findings suggest that CHOP may play a neuroprotective role in hippocampus during ER stress.

CHOP is a downstream component of ER-stress pathways, and its role operates at the convergence of the IRE1, PERK and ATF6 pathways. ER stress induces stronger CHOP response than DNA damage does [12]. CHOP protein overexpression will induce cell apoptosis, a process which can be inhibited by BCl-2 [9]. CHOP could also work as a transcriptional factor that regulates genes involved in either survival or death [14]. The phenotype of CHOP knockout mice has been shown to possess a normal development and normal fertility, implicating that CHOP is dispensable for organogenesis and development [12], [15]. Recently, Miyazaki and colleagues investigated the pathological role of CHOP in myocardial reperfusion injury using a CHOP knockout mouse model. They found that an ER stress-induced CHOP-mediated pathway, which is activated in part by superoxide overproduction after reperfusion, is involved in the myocardial ischemia/reperfusion injury by inducing cardiomyocyte apoptosis and myocardial inflammation [16]. In contrast, Halterman and colleagues have recently reported that CHOP deletion enhances the neuronal susceptibility to both hypoxic and thapsigargin-mediated injury and attenuated brain-derived neurotrophic factor-induced neuroprotection [17]. They postulated that the ability of CHOP to induce cellular injury would depend on the balance of adaptive *versus* pathological modifications to the cellular complement of CHOP. In the present study, we chose CHOP−/− mice for studying the role of CHOP in hippocampal cell apoptosis and memory impairment after ER stress induction by tunicamycin, which blocks protein glycosylation. The results showed that CHOP−/− mice treated with tunicamycin show an increased hippocampal neurons and glial cell apoptosis and memory performance impairment as compared with wild type mice.

GRP78 is well-known as an ER chaperone that belongs to the heat-shock protein (HSP) family. It is present in all cells and plays an important role in maintaining cellular homeostasis [18]. The primary functions of GRP78 are : binding to hydrophobic patches on nascent polypeptides in the ER; and acting as one of the initial components of the signaling cascade that produces the UPR [19]. The UPR is initiated by proteins accumulation in the ER lumen, then triggers the activation of the transmembrane protein kinase/endoribonuclease IRE1, the activating transcription factor 6 (ATF6), and the endoplasmic reticulum resident kinase (PERK). IRE1 plays various roles in the ER responses to unfolded proteins (adaptation, alarm, and apoptosis), mainly through its actions upon XBP-1 (adaptation), TRAF2 (alarm), and apoptosis effectors caspase-12 and ASK1 [20]. The function of XBP1 is to act as a transcription factor that activates transcription of genes coding for proteins needed for the ER protein folding and processing reactions to decrease the unfolded proteins accumulation [21]. In addition to kinase activation, IRE-1 can be a target of some of the BCl-2 family of proteins that regulate cell death [22]. The IRE1-ASK1-JNK signalling pathway is also a cell death pathway, because JNK-mediated phosphorylation has been known to activate the pro-apoptotic BCl-2 family member BIM, while inhibiting the antiapoptotic protein BCl-2 [23], [24]. IRE-1 can generate spliced mRNA of the X-box binding protein (XBP-1) through its RNase activity, and then the XBP-1 protein upregulates the expression of GRP78 [18], [25]. Therefore, GRP78 can play dual roles in both initiating UPR and binding to the unfolded protein for further processing during ER stress. Lacour and colleagues utilized organotypic slice of hippocampus to explore the time sequence of GRP78 activation after tunicamycin exposure, and found that the peak of GRP78 activation was 7 days after tunicamycin administration [26]. They also postulated that the elevation of GRP78 levels occurs prior to the neuronal loss, and it might be a neuroprotective mechanism. In the present study, one day after injection of tunicamycin caused the expressions of GRP78 and CHOP in the hippocampus of wild type mice, but CHOP expression was absent in CHOP−/− mice. Moreover, CHOP−/− mice also showed the decreased IRE-1 protein expression after tunicamycin administration. The decreased IRE-1 and XBP-1 in CHOP−/− mice may reflect its decreased ability to cope with ER stress. Although decreased IRE-1 and XBP-1 expressions were observed in CHOP−/− mice, the GRP78 expression remained elevated. We have found that ATF6 expression is elevated in hippocampus of tunicamycin-treated CHOP−/− mice. Thus, the induction of GRP78 expression in CHOP−/− mice may due to the elevated ATF6, which it can contribute to trigger the transcription of GRP78 during ER stress [20]. However, the detail mechanism needs to be clarified in the future. On the other hand, Li and colleagues have recently demonstrated that differences in ER stress kinetics by inducers can result in transient and prolonged JNK activation, and subsequently in cell survival and apoptosis [27]. Due to the normal defense to ER stress in wild type, the ER stress inducer results in “slow motion” to maintain a transient JNK activation, and the results are cell survival. As we mentioned earlier, the defense to ER stress is decreased in CHOP−/− mice, and the ER stress inducer will be deemed “fast motion”. Therefore, prolonged JNK activation and increased apoptosis were observed in CHOP−/− mice.

In conclusion, we demonstrated for the first time that intracerebroventricular injection of tunicamycin triggered ER stress in hippocampus of wild type and CHOP−/− mice. The CHOP−/− mice showed no CHOP expression, decreased IRE-1 and XBP-1 expressions, and increased JNK phosphorylation after tunicamycin administration. CHOP−/− mice also suffered from greater cell apoptosis in hippocampus and worse memory-related performance tests (passive avoidance and water maze tests) when compared with wild type mice. These findings suggest that CHOP may play a neuroprotective role in hippocampus during ER stress. However, the detail mechanisms for impaired memory, such as the roles of UPR, long-term potentiation (LTP), calcium signaling, and memory-related proteins, need to be clarified in the future.
